# Network meta-analysis of deaths from various underlying diseases after COVID-19 infection

**DOI:** 10.3389/fpubh.2022.959073

**Published:** 2022-08-25

**Authors:** Jinqiang Yang, Ming Li, Renmin Zhang

**Affiliations:** ^1^Department of Clinical Medicine, Jinzhou Medical University, Jinzhou, China; ^2^Department of Clinical Medicine, Dalian Medical University, Dalian, China; ^3^Department of Postgraduate Base of Weihai Central Hospital of Jinzhou Medical University, Weihai, China

**Keywords:** novel coronary pneumonia, death factors of new coronary pneumonia, new coronary pneumonia complications, prognosis of novel coronavirus, COVID-19 with underlying diseases

## Abstract

Network meta-analysis of deaths from various underlying diseases after COVID-19 infection. This study included more than 10 research centers with the same level of care. In total, 1,676 subjects were included in our study, including 1,122 men and 554 women, patients diagnosed with COVID-19, and combined with underlying diseases; provided data on the number of deaths from related diseases, such as hypertension, diabetes, heart disease, cerebrovascular disease, malignant tumor, chronic kidney disease, chronic liver disease, and respiratory disease. The comparison RR between hypertension and different diseases shows that it is (RR = 2.35, 95% CI: 1.47, 3.98) compared with diabetes, compared with coronary heart disease (RR = 2.57, 95% CI: 1.5, 4.4), compared with cerebrovascular disease (RR = 3.68, 95% CI: 1.87, 7.29), compared with malignant tumor (RR = 6.35, 95% CI: 3.45, 11.97), and compared with chronic kidney disease (RR = 5.53 95% CI: 3.04, 10.34), compared with chronic liver disease (RR = 15.51, 95% CI: 5.26, 50.98), compared with respiratory diseases (RR = 4.35, 95% CI: 2.37, 7.65), RR values are >1, which is statistically significant. The surface under the cumulative ranking curve (SUCRA) showed that the ranking of disease mortality from high to low was hypertension> diabetes> heart disease> cerebrovascular disease> respiratory disease> chronic kidney disease> malignant tumor> chronic liver disease. The study that hypertension, diabetes, and heart disease are the top three risk factors for patients infected with COVID-19, and management of these patients should be strengthened to improve the prognosis of patients. Ethical approval and patient consent are not required as this study is a meta-analysis based on published studies. The results of this network meta-analysis will be submitted to a peer-reviewed journal for the publication.

## Introduction

So far in the 21st century, human beings have witnessed deadly pandemics related to novel coronaviruses, including SARS, Middle East Respiratory Syndrome (MERS), and COVID-19. All these viruses that cause acute respiratory infections are highly contagious in nature and/or cause high mortality (1). The recent outbreak of COVID-19, caused by a highly contagious coronavirus known as severe acute respiratory syndrome coronavirus 2 (SARS-CoV-2), is still spreading globally as of the preparation of the manuscript, and the number of cases and deaths continue to rise (2). It is well-known that this pandemic has brought huge challenges to the medical system and social economy of various countries, and poses a major threat to global human health. COVID-19 is considered to be one of the largest global public health crises since the 1918 influenza pandemic (3).

Although the gradual popularization of vaccines has a positive effect on the prevention and control of the spread of COVID-19, it still cannot prevent the occurrence of new deaths, with millions of related deaths reported at the time of preparation of the manuscript (4, 5). The clinical manifestations of COVID-19 include asymptomatic carriers and explosive diseases characterized by acute respiratory failure. Studies have found that patients infected with COVID-19 have a high risk of acute respiratory distress syndrome (ARDS) or multiple organ failure, with approximately 5% of infected patients and 20% of hospitalized patients developing severe symptoms requiring intensive care (6, 7). Some epidemiological studies have shown that advanced age and/or a history of serious underlying diseases, such as cardiovascular and cerebrovascular diseases, are associated with the mortality rate of COVID-19 (8). In addition, it has been reported that the high mortality may be attributed to virus-activated “cytokine storm syndrome” (9). Bienvenu et al. (10) conducted a study on gender differences in immune response and cardiovascular comorbidities, and found that the male patients infected with COVID-19 had a higher mortality rate. Yang et al. (7) conducted a retrospective analysis of 710 patients in the early stage of the COVID-19 outbreak and found that elderly patients with comorbidities and ARDS had an increased risk of death. Other studies have reported risk factors for death in patients with COVID-19 that advanced age, men, hypertension, cardiovascular disease, diabetes, chronic obstructive pulmonary disease, and malignant tumors are related to a higher risk of death in patients with COVID-19 infection (8, 11–13). A meta-analysis of 42 studies showed that compared with children without underlying diseases, children with COVID-19 infections with comorbidities were at higher risk of associated death (14).

Since the outbreak of the COVID-19 pandemic, a large number of studies have reported risk factors related to severe disease or mortality rates, and the combination of underlying diseases in infected persons has been considered to be associated with mortality. However, there have been no systematic reports of related deaths among COVID-19 infected persons under various underlying diseases. Among the factors related to the mortality rate of COVID-19, the specific risk of death of infected persons under various underlying diseases is not clear.

In addition, the incidence rate of chronic diseases in each country is different, and the population of each country is also different from the target population of this study of chronic diseases. The purpose of this article is to calculate the death rate of novel coronavirus under different chronic diseases through scientific statistical methods, establish a network diagram through mesh meta-analysis, fit the model, and eliminate heterogeneity. Calculate the mortality of patients with novel coronavirus under different basic diseases.

Network meta-analysis is a relatively new statistical method, which can be combined and ranked based on the effect of multiple interventions and risk factors. Exclude the heterogeneity of various articles, so as to select the best intervention measures and risk factors. In order to better solve the heterogeneity of risk factors in various regions and articles, observational mesh meta-analysis has emerged as a more novel statistical method, which is divided into the Bayesian school and frequency school. This article creatively uses observational mesh meta-analysis to explore the differences in mortality under various basic diseases of novel coronavirus. Excluding the different incidence rate and heterogeneity of articles in various regions, we finally come to the basic disease that has the highest impact on the mortality of novel coronavirus.

## Methods

### Design

A network meta-analysis using a Bayesian framework will be implemented in this study. This protocol of network meta-analysis will be performed on the basis of the Preferred Reporting Items for Systematic review and Meta-Analysis Protocol (PRISMA-P), and the reporting of the following network meta-analysis will obey the PRISMA extension statement for reporting of systematic reviews incorporating network meta-analysis of healthcare interventions. In this article, Bayesian mesh meta-analysis method is used. First, the whole document screening process is made, and the flow chart is drawn, and the basic information of different documents is drawn, eliminating unsuitable and low-quality literature, determining the final inclusion of literature and extracting relevant data. Second, the mesh relationship diagram is drawn through the model, and the action size of each risk factor is clarified through the network relationship diagram. Different network diagrams and the size of circles in the diagram represent different relationships between different basic diseases. The effect of Bayesian model is evaluated by using the trajectory diagram and density diagram. Only when there is a good fitting, convergence, and diagnostic diagram can the calculation results of this model be used and the RR value is taken to compare each risk factor in pairs, and the League map is drawn. Through the RR value, the maximum influencing factor is found. The influence probability of each risk factor is calculated by the Bayesian model, and finally, the value of this study is evaluated by the heterogeneity test. We searched seven databases, namely, PubMed, Embase, Cochrane, Google Scholar, Wanfang, VIP, and CNKI, and the search time was set from 1 January 2020 to 12 December 2021. The keywords used in our search include COVID-19, mortality, death, novel coronavirus, novel coronary pneumonia, death factors, death, and comorbidities.

### Patient and public involvement

Patients were not involved in the design, conduct, reporting, or dissemination plans of this research.

### Inclusion and exclusion criteria

The inclusion criteria in the study were: patients diagnosed with COVID-19 and combined with underlying diseases, provided data on the number of deaths from related diseases, no restriction on the type of study. The exclusion criteria were: animal experiments; duplicate studies; meta-analyses, reviews, guidelines, and letters.

### Data collection and quality evaluation and statistical analysis

Data were extracted by two independent investigators in accordance with pre-determined information extraction tables (first author, publication year, study type, location, sample size, age, gender, disease, and the number of disease-related deaths). Any differences were negotiated and resolved with a third investigator.

**Figure 1:**
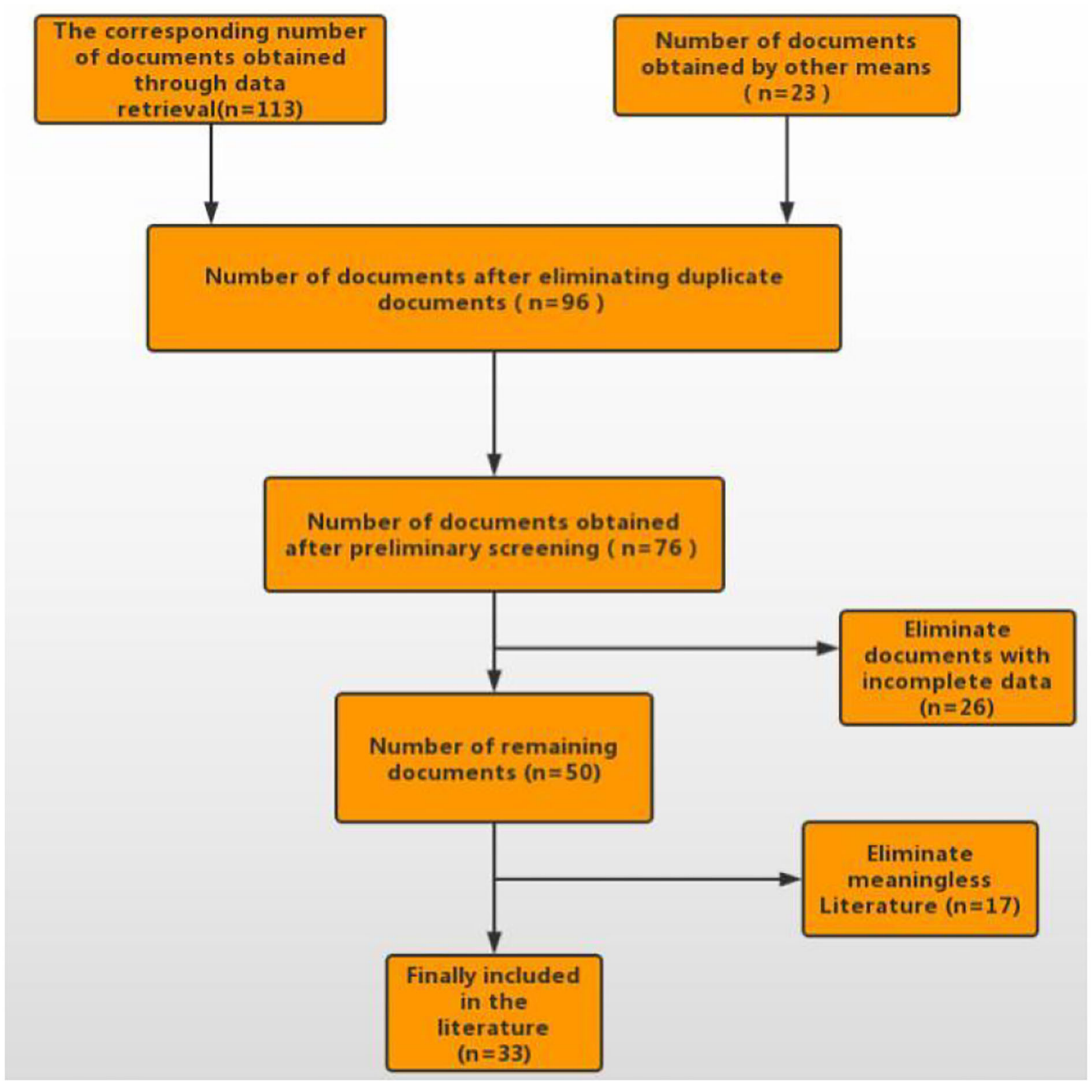
Mesh meta-analysis flow chart.

A total of 113 articles were retrieved from the databases, and 23 articles were obtained through other means. After removing duplicates, 96 articles were obtained and 76 of them were left after the preliminary screening of titles and abstracts. We read the remaining 76 articles in full text and finally determined to include 33 articles (see Figure 1).

The Newcastle–Ottawa Scale (NOS) was used to assess the quality of the cohort studies, which was divided into eight items in three blocks, namely, selection, comparability, exposure assessment or outcome assessment. The total score of NOS in cohort studies is nine points, and a score of ≥6 is generally considered as a high-quality study. The American Institute for health care quality and Research (AHRQ) is a quality evaluation of cross-sectional studies, including 11 items, which are answered with “yes,” “no,” and “unclear,” respectively. If the answer is “no” or “unclear,” the score of this item is 0; If the answer is “yes,” the score of this item is 1 point. Evaluation criteria: (1) low quality 0–3 points; (2) Medium quality 4–7 points; (3) High quality 8–11 points. Gemtc 0.8 program package of R 4.0 software and JAGS software were adopted combined with the Markov Chain Monte Carlo (MCMC) method to conduct Bayesian network meta-analysis. Relative risk (RR) was used as the effect size for binary data, and the 95% prediction interval (CI) was calculated. In total, three Markov chains were selected for the initial value setting, and the total number of iterations was set at 10,000. The first 6,000 annealing times were used to eliminate the influence of the initial value, and sampling started after 6,001 times. Iterative convergence was determined by calculating the potential scale reduction factor (PSRF) and the Gelman Rubin Brooks diagnosis method, and the convergence was better with the reduction factor tending to 1. For the closed loop, the node analysis model was used to detect the inconsistency. *P* ≥ 0.05 obtained from the analysis by node splitting method indicated no significant inconsistency, and the consistency model would be used for analysis. The rank probability plot was employed to rank the mortality of different diseases. Direct and indirect comparisons between different diseases were presented by drawing a network diagram, and publication bias was tested using an adjusted funnel plot.

## Results

### Characteristics of the included studies

The characteristics of the included 33 articles are shown in Table 1. The studies, all published in 2020–2021, were conducted in China, Japan, South Korea, Iran, India, and Kuwait, with a major focus on China. A total of 1,676 patients were enrolled in the included studies, including 1,122 men and 554 women. Table 1 lists the detailed information of each article. The NOS scores of the included 33 articles were all ≥6 points, indicating good research quality. The AHRQ scores of the included 33 articles were all ≥4 points, indicating that the quality of the literature source is acceptable.

**Table 1:** Basic information of included literature.

**Inclusion time**	**First author**	**Place**	**Number of samples**	**Data type**	**Average age**	**Male: Female**
2020	Min Li	China	12	Retrospective study	68.2	10:2
2020	Wei Tan	China	63	Retrospective study	71	45:18
2020	Kai Hu	China	42	Retrospective study	51.2	29:13
2020	Na Wang	China	15	Retrospective study	62.2	9:6
2020	Bing Su	China	188	Cohort study	71.5	105:83
2020	Hai Chao Liu	China	13	Retrospective study	74	10:3
2020	Jian Hua Sun	China	110	Retrospective study	68	65:45
2020	Yi Hu	China	52	Retrospective study	70.7	29:23
2020	Wei Xiong	China	31	Retrospective study	72	22:9
2020	Bing Wang	China	46	Cohort study	68.7	28:18
2020	Hui Zhu	China	37	Retrospective study	76.3	26:11
2020	Kai Dai	China	49	Retrospective study	72.8	32:17
2020	Mariam Ayed	Kuwait	47	Cohort study	70.2	42:5
2020	Fei Tong	China	54	Retrospective study	69	38:16
2020	Lu	Japan	23	Retrospective study	80	15:8
2020	Mohamad Nikpouraghdam	Iran	239	Retrospective study	70	176:72
2020	Ji Yeon Lee	Korean	20	Retrospective study	77	14:6
2020	Wei Jie Guan	China	50	Cohort study	80	32:18
2020	Anirban Gupta	India	49	Cohort study	78	36:13
2021	Lin Jun Li	China	24	Retrospective study	83	17:7
2021	Wei Zhang	China	30	Retrospective study	72	17:13
2021	Fei Xiao	China	53	Retrospective study	74	37:16
2021	Yong Le Yuan	China	52	Retrospective study	76	33:19
2021	Wen Feng Lu	China	28	Cohort study	69	20:8
2021	Hai Chao Liu	China	13	Retrospective study	77	10:3
2021	Ling Lu	China	33	Cohort study	73	25:8
2021	Anyaypoma-Ocón W	Spain	133	Retrospective study	82	96:37
2021	Hui Zhu	China	37		77	27:10
2021	Li Hong Chi	China	18	Retrospective study	87	10:8
2021	Xi Liu	China	47	Retrospective study	68	32:15
2021	Qian Lu	China	73	Retrospective study	67	40:33
2021	Wei Song	China	11	Cohort study	70	6:5
2021	Min Li	China	12	Retrospective study	68	10:2
2021	Juan Yang	China	31	Retrospective study	78	20:11
2021	Lin Jun Li	China	24	Retrospective study	88	16:8

### Network analysis

#### Evidence relationship of the included studies

**Figure 2:**
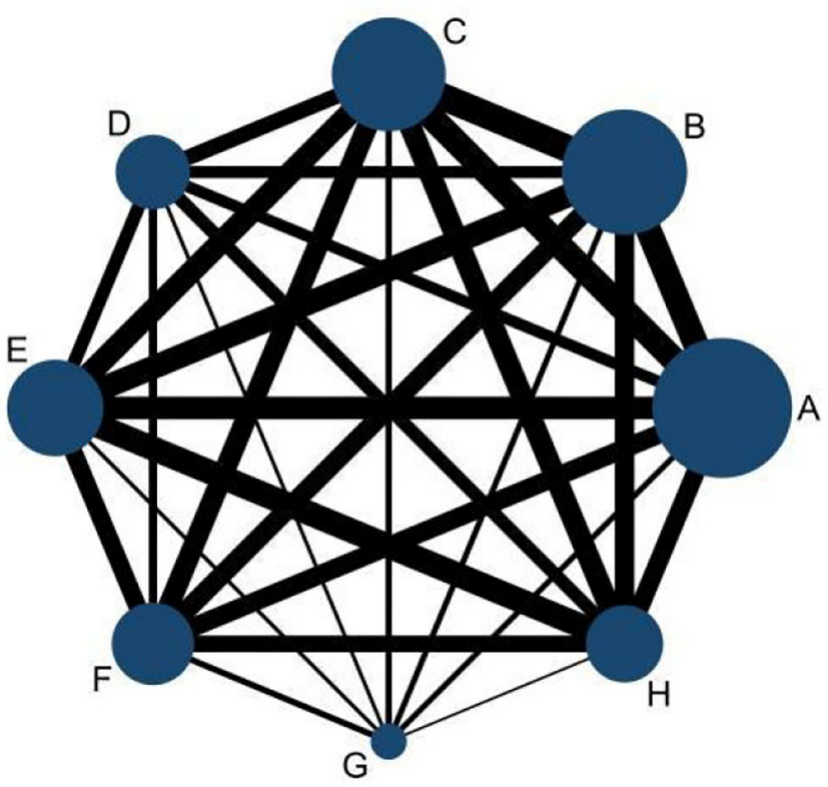
Network diagram of comparison of different diseases. A, hypertension; B, diabetes; C, heart disease; D, cerebrovascular disease; E, malignant tumor; F, chronic kidney disease; G, chronic liver disease; H, respiratory disease.

**Figure 3:**
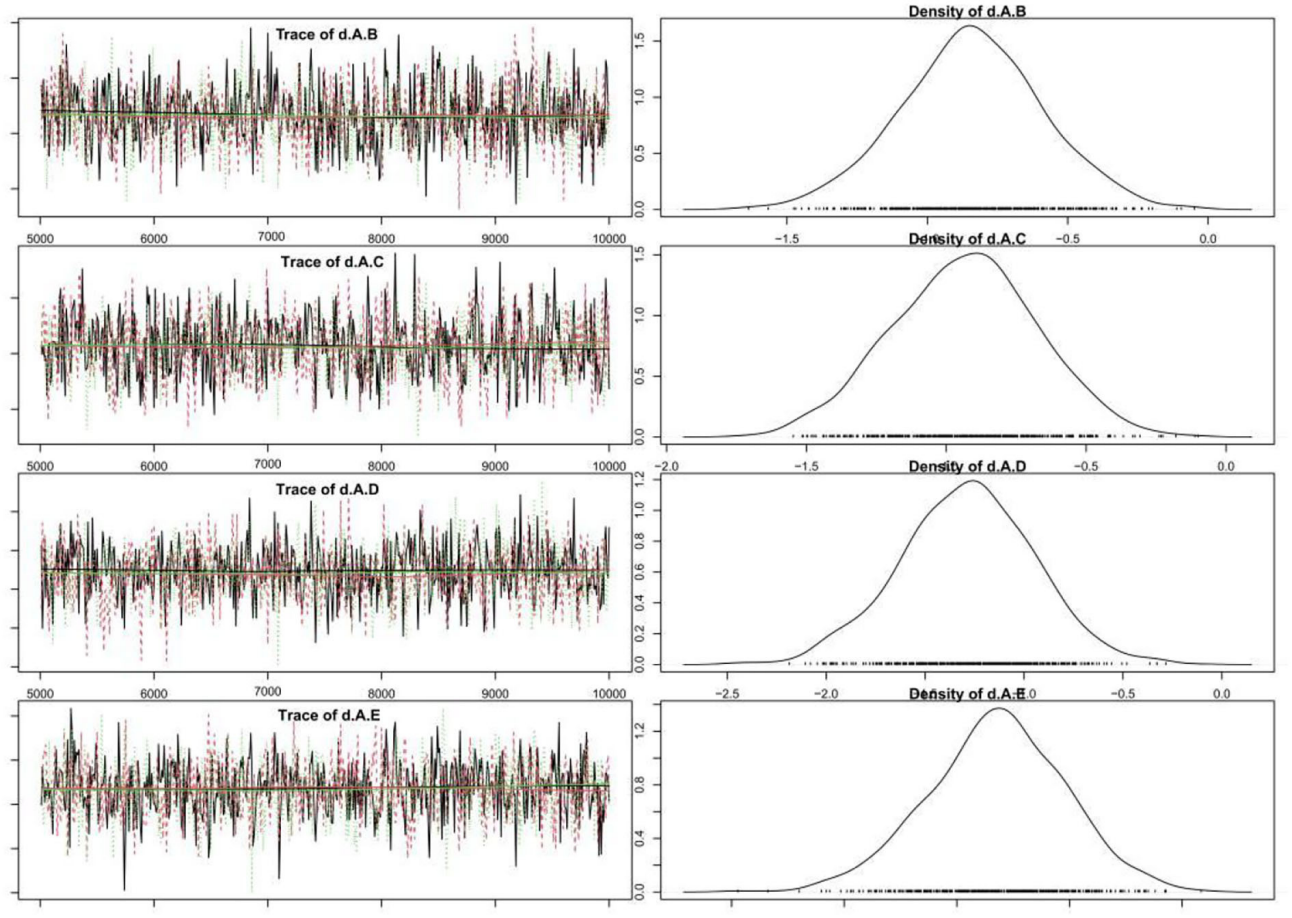
Trajectory diagram and density diagram.

A total of 33 studies reported the mortality of underlying diseases, and the network meta-analysis involved eight diseases, namely, hypertension, diabetes, coronary heart disease, cerebrovascular disease, malignant tumor, chronic kidney disease, chronic liver disease, and respiratory disease. The reticular relationship of mortality of different underlying diseases complicated with COVID-19 is shown in Figure 2. The larger the circle in the figure is, the more patients with the disease are; the thicker the straight line is, the more studies on mortality comparison between the two diseases are. The results of the node splitting method showed *P*>0.05, so we chose the consistency model for subsequent network analysis. It can be seen from the trajectory diagram and density diagram that the MCMC chain of the model fits well, the fluctuation of a single chain cannot be recognized by the naked eye, and the convergence degree is good. The bandwidth value and calculation result of the density diagram are obviously small, and the curve is stable and smooth, which also proves that the model fits well, which is shown in the figure below Figure 3. The diagnosis of convergence found that PSRF tended to 1, reflecting good convergence.

The relative mortality results of different diseases are presented in Table 2 in the form of a league table. In the comparison of the proportion of dead patients in the total dead patients under different basic diseases through the League chart, the comparison RR between hypertension and different diseases shows that it is (RR = 2.35, 95% CI: 1.47, 3.98) compared with diabetes, compared with coronary heart disease (RR = 2.57, 95% CI: 1.5, 4.4), compared with cerebrovascular disease (RR = 3.68, 95% CI: 1.87, 7.29), compared with malignant tumor (RR = 6.35, 95% CI: 3.45, 11.97), and compared with chronic kidney disease (RR = 5.53 95% CI: 3.04, 10.34), compared with chronic liver disease (RR = 15.51, 95% CI: 5.26, 50.98), compared with respiratory diseases (RR = 4.35, 95% CI: 2.37, 7.65), RR values are >1, which is statistically significant, suggesting that the mortality of patients with essential hypertension is greater than other diseases. On the contrary, the RR values of different basic diseases compared with hypertension showed that diabetes compared with hypertension (RR = 0.53, 95% CI: 0.25, 0.68), coronary heart disease compared with hypertension (RR = 0.53, 95% CI: 0.25, 0.68), cerebrovascular disease compared with hypertension (RR = 0.53, 95% CI: 0.25, 0.68), malignant tumor compared with hypertension (RR = 0.53, 95% CI: 0.25, 0.68), chronic kidney disease compared with hypertension (RR = 0.53, 95% CI: 0.25, 0.68), compared with chronic liver disease and hypertension (RR = 0.53, 95% CI: 0.25, 0.68), respiratory disease and hypertension (RR = 0.53, 95% CI: 0.25, 0.68), all combined effect values RR are <1, which once again proves that patients with basic diseases of hypertension account for the first proportion of death.

**Table 2:** Results of network analysis of different diseases.

A	**A**	0.53 (0.25, 0.68)	0.49 (0.23, 0.67)	0.367 (0.14, 0.53)	0.20 (0.08, 0.29)	0.13 (0.1, 0.33)	0.04 (0.02, 0.19)	0.27 (0.13, 0.42)
B	2.35 (1.47, 3.98)	B	0.90 (0.54, 1.5)	0.60 (0.33, 1.25)	0.36 (0.2, 0.69)	0.41(0.23, 0.8)	0.16 (0.05, 0.45)	0.50 (0.3, 0.99)
C	2.57 (1.5, 4.4)	1.07 (0.67, 1.85)	C	0.7 (0.30, 1.4)	0.40 (0.21, 0.74)	0.45 (0.25, 0.85)	0.13 (0.05, 0.48)	0.55 (0.32, 1.11)
D	3.68 (1.87, 7.29)	1.53 (0.8, 3.07)	1.43 (0.71, 2.73)	D	0.54 (0.27, 1.19)	0.65 (0.32, 1.37)	0.32 (0.07, 0.76)	0.80 (0.41, 1.73)
E	6.35 (3.45, 11.97)	2.71 (1.44, 5.03)	2.46 (1.34, 4.81)	1.76 (0.84, 3.72)	E	1.17(0.57, 2.36)	0.50 (0.12, 1.23)	1.43 (0.76, 2.88)
F	5.53 (3.04, 10.34)	2.34 (1.25, 4.31)	2.14 (1.18, 4.01)	1.52 (0.73, 3.08)	0.87 (0.42, 1.76)	F	0.36 (0.1, 1.1)	1.25 (0.66, 2.44)
G	15.51 (5.26, 50.98)	6.69 (2.23, 21.58)	6.19 (2.09, 20.33)	4.36 (1.32, 14.95)	2.52 (0.81, 8.53)	2.87 (0.91, 10.07)	G	3.60 (1.13, 12.9)
H	4.35 (2.37, 7.65)	1.82 (1.01, 3.29)	1.69 (0.9, 3.15)	1.18 (0.58, 2.44)	0.68 (0.35, 1.32)	0.78 (0.41, 1.51)	0.28 (0.08, 0.89)	H

A, hypertension; B, diabetes; C, heart disease; D, cerebrovascular disease; E, malignant tumor; F, chronic kidney disease; G, chronic liver disease; H, respiratory disease.

**Figure 4:**
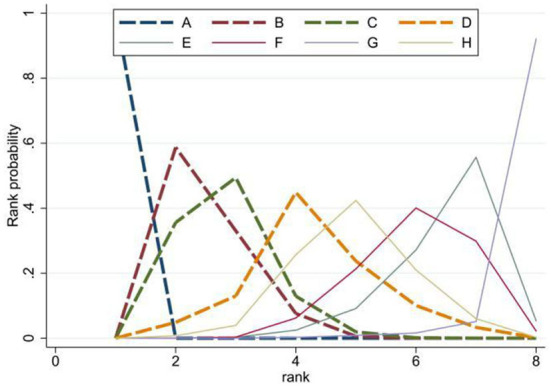
Rank probability of the mortality of different diseases.

**Figure 5:**
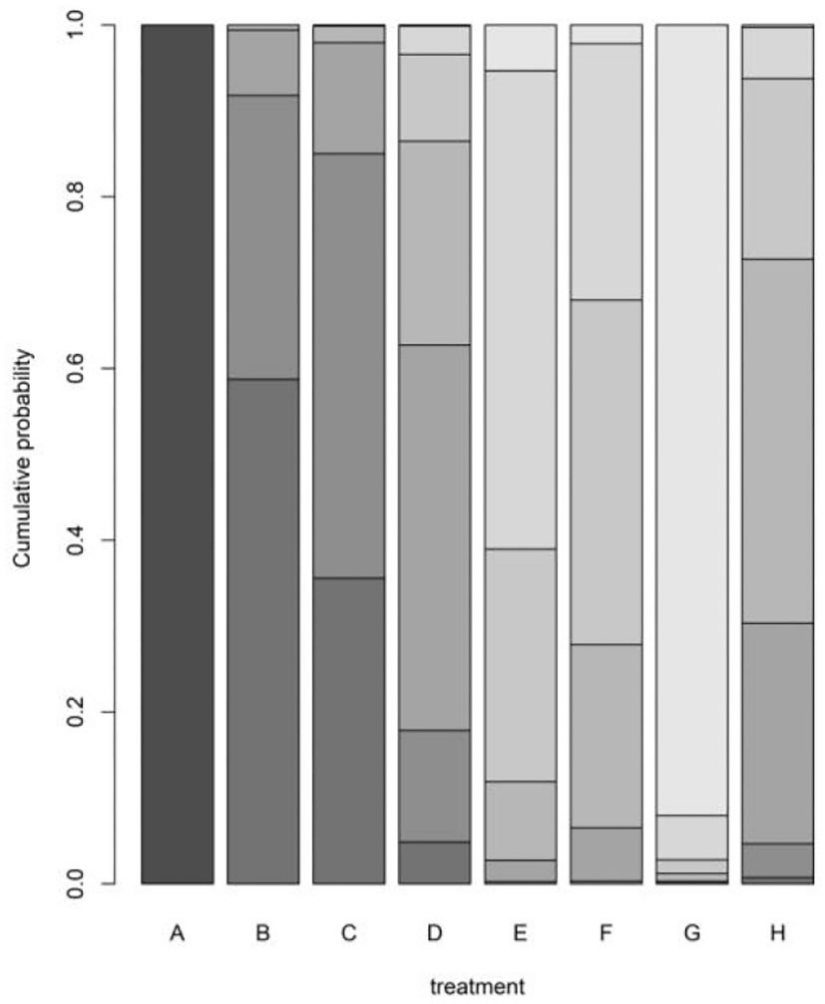
Probability diagram.

**Figure 6:**
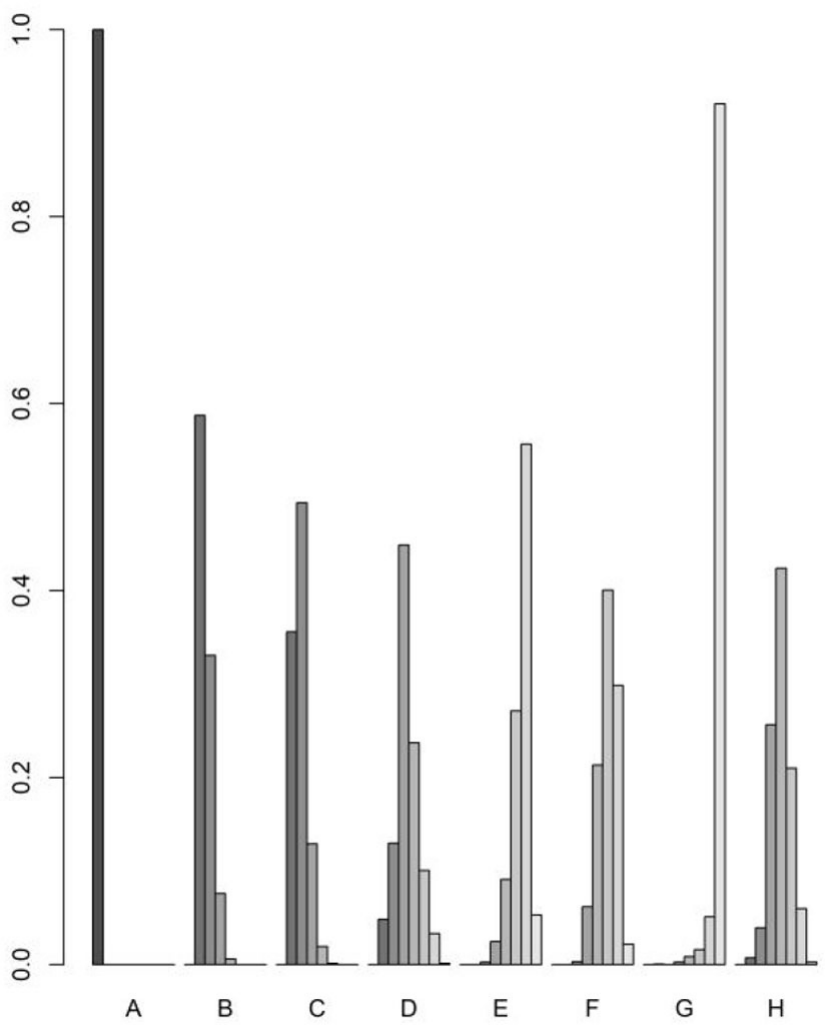
Cumulative probability diagram.

The rank probability of the mortality of different diseases is shown in Table 3. The results showed that the ranking of disease mortality, from high to low, was hypertension (100%)> diabetes (59%)> heart disease (49%)> cerebrovascular disease (45%)> respiratory disease (42%)> chronic kidney disease (40%)> malignant tumor (56%)> chronic liver disease (92%). We draw the probability diagram of the two sorting probabilities, as shown in the figure below Figure 4. Moreover, through the probability diagram and the probability diagram of involvement, it can be seen that hypertension has a significant advantage in all basic diseases (see Figures 5, 6).

**Table 3:** Ranking probability of different diseases.

**Rank**	**A**	**B**	**C**	**D**	**E**	**F**	**G**	**H**
Rank 1	1.00	0.00	0.00	0.00	0.00	0.00	0.00	0.00
Rank 2	0.00	0.59	0.36	0.05	0.00	0.00	0.00	0.01
Rank 3	0.00	0.33	0.49	0.13	0.00	0.00	0.00	0.04
Rank 4	0.00	0.08	0.13	0.45	0.02	0.06	0.00	0.26
Rank 5	0.00	0.01	0.02	0.24	0.09	0.21	0.01	0.42
Rank 6	0.00	0.00	0.00	0.10	0.27	0.40	0.02	0.21
Rank 7	0.00	0.00	0.00	0.03	0.56	0.30	0.05	0.06
Rank 8	0.00	0.00	0.00	0.00	0.05	0.02	0.92	0.00

A, hypertension; B, diabetes; C, heart disease; D, cerebrovascular disease; E, malignant tumor; F, chronic kidney disease; G, chronic liver disease; H, respiratory disease.

## Discussion

The COVID-19 pandemic poses a major threat to human health on a global scale,as multiple systems can be affected after infection. With the accumulation of epidemic prevention experience, vaccination, and popularization of epidemic prevention knowledge (15), the cure rate of COVID-19 has increased from 5.80% on 2 March 2020 to the current 74.59%, and the cure rate in China has reached 96.7%. However, some patients still died after being infected with COVID-19. Studies have shown that patients infected with COVID-19 with some underlying diseases are more likely to develop secondary serious complications, aggravate the severity of the disease, and increase the fatality rate (16). Therefore, it is necessary to clarify the impact of different underlying diseases on the prognosis of people infected with COVID-19. The current study conducted a network meta-analysis and found that the risk of death after COVID-19 infection in patients with underlying diseases from high to low was: hypertension > diabetes > heart disease > cerebrovascular disease > respiratory disease > chronic liver disease > malignant tumor > chronic liver disease. Through bibliometric statistics, Xie Xingxing et al. found that hypertension, diabetes, and coronary heart disease were the top three with a high risk of death after COVID-19 infection, which is consistent with the results of this study (17). The prevalence rates of hypertension and diabetes in Chinese adults are 23.2% (18) and 10.9% (19), respectively, and combined hypertension and diabetes are the primary and secondary risk factors for the poor prognosis of COVID-19.

This study found that hypertension is the leading risk factor for the death of people infected with COVID-19. Yin et al. (20) retrospectively analyzed 1,580 cases of COVID-19 in Jingzhou City, and found that age >60 years old, combined with hypertension, and shortness of breath at the time of admission were risk factors for death of COVID-19-infected patients, and the poor prognosis of patients with hypertension was 2.004 times that of patients without hypertension. Some studies have suggested that SASR-COV-2 can enter cells through angiotensin-converting enzyme 2 (21), and the administration of ACEI or ARB antihypertensive drugs may enhance the expression of ACE on the cell surface, thus, providing a basis for SARS-CoV-2 to enter cells (22, 23). Liu Qing et al. (24) found no association between ACEI/ARB use and the occurrence of COVID-19 by matching COVID-19 propensity scores in patients with ACEI or ARB in a large UK primary care database (Health Improvement Network). Studies have found that the level of ACE2 decreases in hypertensive patients. Infection with SARS-CoV-2 depletes residual ACE2 in the body, leading to increased angiotensin II levels and promoting the development of acute respiratory distress syndrome (25). At present, the mechanism of poor prognosis in patients with hypertension remains unclear. It is currently considered that there is no need for drug adjustment in patients with COVID-19 taking ACEI/ARB. More and more cases have been reported. The study on the clinical characteristics of COVID−19 shows that hypertension accounts for a significant proportion of deaths in patients with different basic diseases. Moreover, at present, novel coronavirus is highly infectious, the prognosis is general, and there is no specific drug treatment. When facing patients with novel coronavirus with different basic diseases, it suggests that the prognosis of patients with COVID−19 with hypertension and diabetes (26), and coronary heart disease is poor, more timely treatment and help should be given. Moreover, the population of chronic diseases, the incidence rate of chronic diseases, the population of chronic diseases, the prevention policies for COVID−19, and the treatment level of novel coronavirus are different in different countries, so the mortality of novel coronavirus under different basic diseases is different (27). This study calculates the mortality of novel coronavirus under different basic diseases through a scientific and systematic method.

Based on the literature retrieval results, the current study is the first network meta-analysis to explore the correlation between various underlying diseases and COVID-19 deaths. Nevertheless, this study has the following limitations: first, this study included studies from China, Japan, South Korea, Iran, India, and Kuwait. There are differences in epidemic prevention policies, medical resources, and allocation of medical resources in different countries, which will lead to differences in epidemic prevention and control efficiency, treatment rate, and mortality rate (28). In this study, literature screening was strictly conducted according to inclusion and exclusion criteria to reduce potential heterogeneity. The funnel plot did not indicate publication bias, but the results still need to be further verified. Second, all included studies are retrospective, with a lack of prospective studies. In this study, literature of low quality was excluded through literature quality evaluation. Third, the sample size of this study is relatively limited, and the study needs to be updated in the future to further verify the results of this study (29). Fourth, since most of the included literature did not classify diabetes, and considering the high proportion of type-2 diabetes in the population, this study did not determine whether type-1 diabetes affects the prognosis of patients with COVID-19.

## Conclusion

Combined hypertension, diabetes, and heart disease are the top three high-risk factors for the poor prognosis of patients infected with COVID-19, and management of these patients should be strengthened to improve the prognosis of patients. The results of this study need to be further verified by studies with a larger sample size. Through this study, we can know the mortality of COVID-19 under different basic diseases. From the basic level, it can provide direction for the basic research on the prognosis of novel coronavirus, and explore the pathological mechanism of novel coronavirus and hypertension. With the current large-scale vaccination, the reduction of adverse prognostic events of the novel coronavirus, and the emergence of new novel coronavirus strains, we can see that more energy can be put into financial and material resources which are put into the study of clinical manifestations and prognosis of different strains of novel coronavirus.

## Data availability statement

The original contributions presented in the study are included in the article/supplementary material, further inquiries can be directed to the corresponding author.

## Author contributions

JY and ML drafted the manuscript. RZ revised the manuscript critically for important intellectual content. All authors gave final approval of the version to be published and agreed to be accountable for all aspects of the work in ensuring that questions related to the accuracy or integrity of any part of the work are appropriately investigated and resolved, made substantial contributions to the conception and design of the work, and the acquisition, analysis, and interpretation of data for the work.

## Conflict of interest

The authors declare that the research was conducted in the absence of any commercial or financial relationships that could be construed as a potential conflict of interest.

## Publisher's note

All claims expressed in this article are solely those of the authors and do not necessarily represent those of their affiliated organizations, or those of the publisher, the editors and the reviewers. Any product that may be evaluated in this article, or claim that may be made by its manufacturer, is not guaranteed or endorsed by the publisher.
